# Selective targeting of KRAS-Mutant cells by miR-126 through repression of multiple genes essential for the survival of KRAS-Mutant cells

**DOI:** 10.18632/oncotarget.2284

**Published:** 2014-07-31

**Authors:** Toshifumi Hara, Matthew F. Jones, Murugan Subramanian, Xiao Ling Li, Oliver Ou, Yuelin Zhu, Yuan Yang, Lalage M. Wakefield, S. Perwez Hussain, Jochen Gaedcke, Thomas Ried, Ji Luo, Natasha J. Caplen, Ashish Lal

**Affiliations:** ^1^ Regulatory RNAs and Cancer Section, Genetics Branch, Center for Cancer Research, National Cancer Institute, National Institutes of Health, Bethesda, MD, USA; ^2^ Gene Silencing Section, Genetics Branch, Center for Cancer Research, National Cancer Institute, National Institutes of Health, Bethesda, MD, USA; ^3^ Molecular Genetics Section, Genetics Branch, National Cancer Institute, National Institutes of Health, Bethesda, MD, USA; ^4^ Cancer Biology of TGF-beta Section, Laboratory of Cancer Biology and Genetics, Center for Cancer Research, National Cancer Institute, National Institutes of Health, Bethesda, MD, USA; ^5^ Pancreatic Cancer Unit, Laboratory of Human Carcinogenesis, National Cancer Institute, National Institutes of Health, Bethesda, MD, USA; ^6^ Department of General and Visceral Surgery, University Medicine Göttingen, Germany; ^7^ Cancer Genomics Section, Center for Cancer Research, National Cancer Institute, National Institutes of Health, Bethesda, MD, USA; ^8^ Cancer Systems Biology Section, Laboratory of Cancer Biology and Genetics, Center for Cancer Research, National Cancer Institute, National Institutes of Health, Bethesda, MD, USA

**Keywords:** Colorectal Cancer, miRNA, miR-126, KRAS, KRAS mutant

## Abstract

MicroRNAs (miRNAs) regulate the expression of hundreds of genes. However, identifying the critical targets within a miRNA-regulated gene network is challenging. One approach is to identify miRNAs that exert a context-dependent effect, followed by expression profiling to determine how specific targets contribute to this selective effect. In this study, we performed miRNA mimic screens in isogenic KRAS-Wild-type (WT) and KRAS-Mutant colorectal cancer (CRC) cell lines to identify miRNAs selectively targeting KRAS-Mutant cells. One of the miRNAs we identified as a selective inhibitor of the survival of multiple KRAS-Mutant CRC lines was miR-126. In KRAS-Mutant cells, miR-126 over-expression increased the G1 compartment, inhibited clonogenicity and tumorigenicity, while exerting no effect on KRAS-WT cells. Unexpectedly, the miR-126-regulated transcriptome of KRAS-WT and KRAS-Mutant cells showed no significant differences. However, by analyzing the overlap between miR-126 targets with the synthetic lethal genes identified by RNAi in KRAS-Mutant cells, we identified and validated a subset of miR-126-regulated genes selectively required for the survival and clonogenicity of KRAS-Mutant cells. Our strategy therefore identified critical target genes within the miR-126-regulated gene network. We propose that the selective effect of miR-126 on KRAS-Mutant cells could be utilized for the development of targeted therapy for KRAS mutant tumors.

## INTRODUCTION

MicroRNAs (miRNAs) are small non-coding RNAs (~ 22 nucleotides) that inhibit mRNA stability and/or translation by binding to the 3' untranslated region (UTR) of target mRNAs [1, 2]. Binding of miRNAs to mRNAs occurs via partial complementarity through the seed region, nucleotides (nts) 2-7 or 2-8 at the 5'end of the miRNA. However, complementarity to the seed region is not a requirement for all instances of miRNA-mediated target gene suppression [3-6]. Each miRNA can inhibit the expression of a gene network to regulate a wide variety of cellular processes. Within these gene networks, a few critical gene targets are likely to play a major role in driving the phenotype regulated by a miRNA. Identifying these critical miRNA-regulated genes is essential in understanding the role of a miRNA in disease pathogenesis and for the development of miRNA therapeutics.

Studies in mouse models and cell lines have demonstrated that some miRNAs including miR-155, miR-21, and the miR-17~92 cluster are oncogenic; others, such as let-7, miR-34a and the miR-15~16 cluster, function as tumor suppressors [7-10]. Recent studies have shown that some miRNAs including miR-29a, miR-146 and miR-200 act as tumor suppressors or as oncogenes depending on the cellular context and such miRNAs are designated as context-dependent miRNAs [11-16]. In contrast to the well-established tumor suppressor or oncogenic miRNAs, it is not clear how many cancer-associated miRNAs have context-dependent effects. Therefore, identifying context-dependent miRNAs and understanding the molecular mechanism underlying their selective effect is essential in uncovering the critical targets hidden in miRNA-regulated gene networks.

Context-dependent activities of miRNAs can occur through several mechanisms, including: (1) single nucleotide polymorphisms (SNPs) in the mRNA or miRNA that destroy or create a miRNA binding site, (2) alternative polyadenylation (APA) that may result in altered miRNA regulation because longer 3'UTRs contain more miRNA binding sites, and as a result, are more sensitive to regulation by miRNAs, and (3) the availability of RNA-binding proteins that inhibit the binding of a miRNA to a target transcript. Evidence supporting these mechanistic scenarios includes, the identification of a SNP associated with increased risk of lung cancer and ovarian cancer, that alters a let-7 miRNA binding site in the 3'UTR of the *KRAS* transcript [17, 18]. Shortening of 3'UTR through APA has been linked to oncogenic transformation due to the loss of repression of let-7 target transcripts such as *DICER1* [19], and the RNA-binding protein Pumilo-1 regulates the expression of p27 mRNA during cell cycle progression by inducing a change in the structure of p27 mRNA that allows miR-221 and miR-222 to efficiently suppress p27 expression [20].

Another mechanism by which a miRNA can act in a context-dependent manner is when its target is essential for the viability of cell-type “A” but not cell-type “B”. For example, in the context of oncogenic KRAS, over-expression of a miRNA in KRAS-Mutant cells and KRAS-Wild-type (WT) cells can impair the viability of KRAS-Mutant cells but not KRAS-WT cells by significantly decreasing the expression of a gene that is essential for the viability of only KRAS-Mutant cells. In this study, we set out to exploit this context-dependent activity of miRNAs by identifying miRNAs that act specifically in the context of the activated KRAS oncogenic signaling pathway. KRAS is a membrane bound GTPase that becomes active in the GTP-bound state and is inactive in the GDP-bound state. Activating mutations in KRAS including G12D and G13D lock KRAS in the GTP-bound, constitutively active state to deregulate multiple downstream pathways resulting in deregulated cell growth, evasion from apoptosis and angiogenesis [21-23]. Activated KRAS signaling is associated with multiple cancer types [22-25], including colorectal cancer (CRC), non-small cell lung cancer (NSCLC) and pancreatic ductal adenocarcinoma (PDAC). Several recent studies have reported loss-of-function screens using either RNAi or small molecules to inhibit the survival of KRAS-Mutant cells but not KRAS-(WT) expressing cells [23, 26-29]. These studies identified several proteins essential for survival of KRAS-Mutant cells but not KRAS-WT cells.

To prevent KRAS-Mutant cells from switching to alternative survival pathways it may be necessary to simultaneously inhibit the expression of several proteins. Here, we conducted miRNA mimic screens in isogenic KRAS-Mutant and KRAS-WT HCT116 cells with the aim of identifying miRNAs that exhibit context-dependent activity. Among the several candidate miRNAs, we focused on miR-126 because (1) miR-126 over-expression selectively impaired the survival of a panel of KRAS-Mutant CRC cell lines, (2) miR-126 inhibited clonogenicity of multiple KRAS-Mutant CRC cell lines, and (3) miR-126 levels were significantly lower in CRC tumors expressing KRAS-Mutant as compared to KRAS-WT. We identified the genes miR-126 regulates in KRAS-WT and KRAS-Mutant cells and found that miR-126 suppresses the expression of multiple genes that are synthetic lethal interactors of mutant KRAS. Our findings suggest that the context-dependent effects of miR-126 in KRAS-Mutant cells could be utilized for the development of a novel targeted therapy for KRAS mutant tumors.

## RESULTS

### Identification of miR-126 as a selective inhibitor of the viability of KRAS-Mutant cells

**Figure 1:**
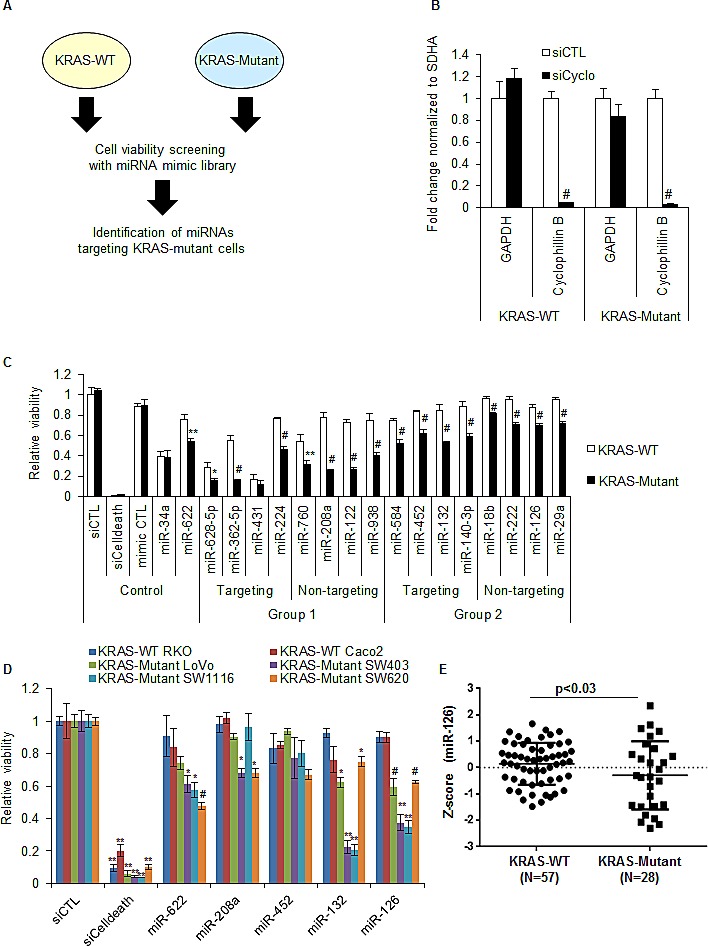
MiR-126 selectively reduces the viability of KRAS-Mutant cells (A) Schematic of the miRNA mimic screening. (B) Isogenic HCT116 KRAS-WT and KRAS-Mutant cells were reverse transfected with a control (siCTL) siRNA or Cyclophilin B siRNAs (siCyclo) for 48 h and RT-qPCR normalized to the housekeeping gene *SDHA* was performed to assess the extent of *Cyclophilin B* knockdown. The housekeeping gene *GAPDH* was used as negative control. (C) The effect of 16 candidate miRNA mimics identified in the initial miRNA mimic screening (Figure S1, Table S1) on cell viability was further validated. The isogenic lines were reverse transfected in 384-well plates with siCTL, siCelldeath or miRNA mimics corresponding to a CTL miRNA (cel-miR-67), miR-34a, miR-622 and each of the 16 candidate miRNAs. MiRNAs were divided into 2 groups based on their expression level in HCT116 cells [31]. Group 1 consists of miRNAs with <100 reads whereas miRNAs with >100 reads were assigned to Group 2. MiRNAs that had at least 1 binding site in the *KRAS* 3'UTR (based on TargetScan predictions) are designated as Targeting; miRNAs that were not predicted by TargetScan to bind the *KRAS* 3'UTR are designated as Non-targeting. (D) A panel of CRC lines expressing KRAS-WT or KRAS-Mutant was reverse transfected with siCTL, siCelldeath, miR-622, miR-208a, miR-452, miR-132, or miR-126 mimics. The effect on cell viability was measured after 72 h using CellTiter-Glo. (E) miR-126 levels were compared between CRC patient samples that were WT or mutant for KRAS. Error bars in B and C represent mean ± standard deviation from 3 independent experiments. *, p<0.05; #, p<0.01; **, p<0.005; ##, p<0.001.

To identify miRNAs that selectively alter the viability of CRC cells harboring mutant KRAS, we decided to perform replica parallel screens (Figure 1A) of synthetic miRNA mimics corresponding to 879 human miRNAs in isogenic HCT116 KRAS-wild-type (KRAS-WT) and KRAS-Mutant (G13D/−) cells [30]. First, we determined the transfection efficiency of KRAS-WT and KRAS-Mutant cells by transfecting the cells with a control siRNA (siCTL) or a cyclophilin B siRNA (siCyclo) for 48 h. We measured knockdown of Cyclophilin B mRNA by RT-qPCR and observed >95% reduction in Cyclophilin B mRNA in the isogenic cell lines (Figure 1B). Next, we performed miRNA mimic transfections for the 879 miRNAs and performed cell viability assays (Cell Titer-Glo) after 72 h; see Figure S1, S2 for screen quality control data. The majority of miRNAs did not significantly alter the viability of either KRAS-WT or KRAS-Mutant HCT116 cells, or modulated the viability of both cell lines similarly (Figure S1 and Table S1). Fifty four miRNAs induced a difference in the viability of KRAS-Mutant cells compared to KRAS-WT cells when the data for the replica screens was considered (>0.5 difference in median normalized Z-scores; Figures S1). In some cases, a miRNA mimic induced a significant reduction (z-score < -1.645) in the viability of KRAS-Mutant cells but had minimal effects on KRAS-WT cells, for example, miR-628-5p and miR-362-5p. Other miRNA mimics slowed the growth of KRAS-Mutant versus KRAS-WT cells, for example, miR-1248 and miR-222.

To confirm the different effects of specific miRNA mimics on the growth of HCT116 KRAS-Mutant and KRAS-WT cells, we selected 16 miRNAs for validation. Analysis of our small RNA sequencing data from HCT116 cells [31] showed that 8 of the selected miRNAs are not expressed in HCT116 cells (< 100 reads, Group 1, Figure 1C), while 8 selected miRNAs are expressed in HCT116 cells (>100 reads, Group 2, Figure 1C). Within each group, 4 miRNAs had at least one predicted binding site (TargetScan) in the *KRAS* 3'UTR, while the others had no computationally predicted sites in the *KRAS* 3'UTR (Figure 1C). For comparison, we also included miR-34a, a growth suppressive miRNA not predicted to target *KRAS* 3'UTR, and the KRAS-targeting miR-622 [32]. Of these 16 miRNAs, 15 decreased the viability of KRAS-Mutant cells when compared to KRAS-WT cells (Figure 1C). As expected, miR-34a reduced the viability of both isogenic lines to the same extent; the KRAS-targeting miR-622 was significantly more potent in KRAS-Mutant cells as compared to KRAS-WT cells. Of note, we observed a modest reduction in the viability of both KRAS-WT and KRAS-Mutant cells (Figure 1C) with CTL (cel-miR-67) mimics. Therefore, for further experiments, we used siCTL instead of CTL mimics.

Next, we transfected 6 additional CRC lines that included 2 that were KRAS-WT (RKO and CaCo2) and 4 that harbor mutations in KRAS (LoVo, SW403, SW1116 and SW620) to determine whether the KRAS mutant-specific effects of these 15 miRNAs extended to other CRC lines. As a positive control, siCelldeath was lethal in all 6 CRC lines; the KRAS-targeting miR-622, reduced the viability of 3 out of 4 CRC lines (Figure 1D). Among the 16 miRNAs, only 2 miRNAs - miR-126 and miR-132, consistently inhibited the viability of at least 3 of the 4 KRAS-Mutant lines, but not the KRAS-WT lines (Figure 1D and S3). MiR-132 is an intergenic miRNA and predicted by multiple alogorithms such as RNA22, TargetScan and RNAHybrid to target the *KRAS* 3'UTR. MiR-126 is embedded in the intron of the protein-coding gene *EGFL7* and is not predicted by TargetScan to bind to the *KRAS* 3'UTR but is predicted by other algorithms such as RNA22. Using a 85 CRC tumor dataset available through the Cancer Genome Atlas (TCGA at cBioPortal) [33] we compared the expression of miR-126 in CRC tumor samples that were KRAS-WT or KRAS-Mutant. We found significantly decreased levels of miR-126 (p<0.03) and *EGFL7* mRNA (p<0.02) in 28 KRAS-Mutant tumors as compared to the 57 KRAS-WT tumors (Figure 1E, S4A and Table S2). A similar analysis performed for miR-132 suggested that miR-132 is not differentially expressed in KRAS-WT and KRAS-Mutant CRC tumors (Figure S4B and Table S2). To determine if the selective effect of miR-126 on KRAS-Mutant cells was not restricted to CRC lines, we next transfected a panel of KRAS-WT (BxPC-3 and Hs 700T) and KRAS-Mutant (Capan-1, Capan-2, PANC-1 and MIA paca-2) pancreatic cancer cell lines with siCTL or miR-126 mimics (20 nM) and measured cell viability after 72 h. We observed significant reduction in the viability of KRAS-Mutant pancreatic cancer lines and almost no effect on KRAS-WT lines (Figure S5), further establishing the selective growth inhibitory effect of miR-126 on KRAS-Mutant cells. As expected, siCelldeath transfections resulted in dramatic reduction in viability irrespective of KRAS mutation status.

Our results suggest that of the 16 miRNAs from the initial screen and from the CRC panel, miR-126 was the only miRNA whose over-expression specifically reduced the viability of HCT116-KRAS-Mutant cells (Figure 1C) and 4 out of 4 KRAS-mutant CRC lines in the CRC panel (Figure 1D). Importantly, miR-126 was significantly down-regulated in KRAS-Mutant tumors as compared to KRAS-WT tumors (Figure 1E). Therefore, it would make sense to reintroduce miR-126 in KRAS-Mutant cells to specifically target KRAS-Mutant cells. We therefore decided to focus on examining the molecular basis of the context-dependent effect of miR-126 observed in KRAS-Mutant cells.

### Selective induction of G1 arrest and inhibition of tumorigenicity by miR-126 in KRAS-Mutant cells

**Figure 2:**
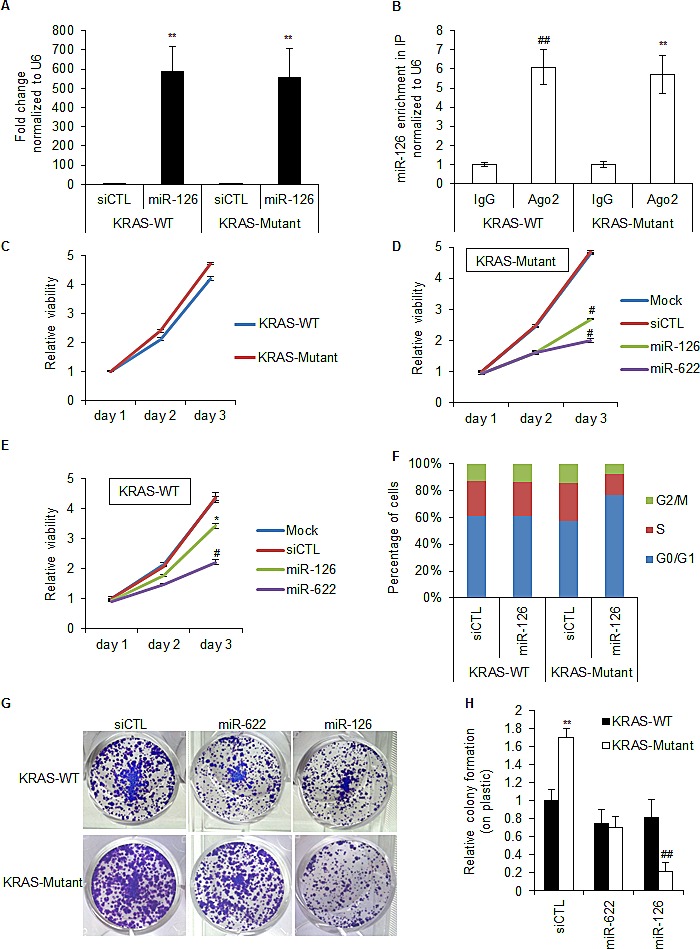
Over-expression of miR-126 increases the G1 compartment in KRAS-Mutant cells (A, B) HCT116 KRAS-WT and isogenic KRAS-Mutant cells were reverse transfected with siCTL or miR-126 mimics for 48 h and the abundance of miR-126 in total RNA (A) and in the IgG or Ago2 IPs (B) performed from cytoplasmic extracts was assessed by RT-qPCR normalized to *U6*. (C-E) Isogenic HCT116 KRAS-WT and KRAS-Mutant cells were reverse transfected with RNAiMAX alone (C), siCTL, miR-622 or miR-126 mimics (D, E) and at the indicated time points the effect on cell viability was measured using Cell counting kit-8. (F) Isogenic HCT116 KRAS-WT and KRAS-Mutant cells were reverse transfected with siCTL or miR-126 mimics for 72 h and the effect on cell cycle progression was analyzed by PI staining. (G and H) The effect of miR-126 on long-term viability of isogenic HCT116 KRAS-WT and KRAS-Mutant cells was assessed by performing colony formation assays on plastic. Cells were reverse transfected with siCTL, miR-622 or miR-126 mimics. After 48 h, 1000 cells were seeded in 12-well plates and stained with crystal violet after 10 days. Representative pictures of cells stained with crystal violet are shown (G) and the results from 3 independent experiments are depicted graphically (H). Error bars represent mean ± standard deviation from 3 independent experiments. *, p<0.05; #, p<0.01; **, p<0.005; ##, p<0.001.

Before studying the biological consequences of miR-126 expression in HCT116 cells in more detail, we wanted to make sure that the differential effect of miR-126 was not due to difference in the magnitude of miR-126 over-expression between the isogenic lines. Indeed, when we transfected the isogenic lines with siCTL or miR-126 mimics for 48 h and measured miR-126 up-regulation by RT-qPCR, we observed almost identical increase in miR-126 expression in the isogenic lines (Figure 2A). Although the extent of miR-126 over-expression in the isogenic lines was identical, it was possible that the selective effect of miR-126 could be due to difference in miR-126 activity. Therefore, we measured miR-126 levels incorporated into the RNA-induced silencing complex (RISC) following transfection. The isogenic KRAS-WT and KRAS-Mutant lines were transfected with siCTL or miR-126 mimics for 48 h and the binding of miR-126 to Ago2, a component of RISC, was determined by RT-qPCR from IgG or Ago2 immunoprecipitates (IP). Introduction of the miR-126 mimics into the isogenic lines resulted in ~6-fold-enrichment of miR-126 in Ago2 IPs in both KRAS-WT and KRAS-Mutant cells suggesting that the activity of the transfected miR-126 was not different between KRAS-WT and KRAS-Mutant HCT116 cells (Figure 2B). This result also suggested that although the miR-126 over-expression was >500-fold, which is typical for miRNA mimic transfections, the amount of miR-126 incorporated into RISC upon transfection with miR-126 mimics is not supraphysiological and is consistent with what we, and others, have recently shown for several miRNAs [31, 34-36]. The results from these control experiments together with our Cyclophilin B knockdown experiments that demonstrate equal transfection efficiency of the isogenic lines (Figure 1A) suggest that the selective effect of miR-126 on KRAS-Mutant HCT116 cells was not due to difference in transfection efficiency or level of miR-126 over-expression/activity in the isogenic lines.

To further investigate the selective effect of miR-126 over-expression on the growth of KRAS-Mutant cells, we examined the viability of these cells over time compared to siCTL and the positive control miR-622. The HCT116 KRAS-WT and KRAS-Mutant cells proliferated at similar rates (Figure 2C). However, introduction of miR-126 was more effective in inhibiting the viability of KRAS-Mutant as compared to KRAS-WT cells (Figure 2D and 2E). To determine whether the selective effect of miR-126 was due to increased apoptosis or cell cycle arrest, we performed flow cytometry analysis after propidium iodide staining of the isogenic lines transfected with siCTL or miR-126 mimics for 48 h. MiR-126 mimics specifically increased the G1 compartment in the HCT116 KRAS-Mutant cells (Figure 2F). We did not observe a sub-G1 peak in these experiments, suggesting that miR-126 does not induce apoptosis in these cells. To assess the long-term effects of miR-126 over-expression, we conducted colony formation assays on plastic, 10 days after transfecting HCT116 KRAS-WT or KRAS-Mutant cells with siCTL or miR-126 mimics. Consistent with the anti-proliferative effect of miR-126 that we had observed by PI staining, we observed decreased colony formation upon over-expression of miR-126 in KRAS-Mutant cells but not in KRAS-WT cells (Figure 2G and 2H). These results suggest that miR-126 selectively induces growth arrest in HCT116-KRAS-Mutant cells and that this phenotype was sustained.

**Figure 3:**
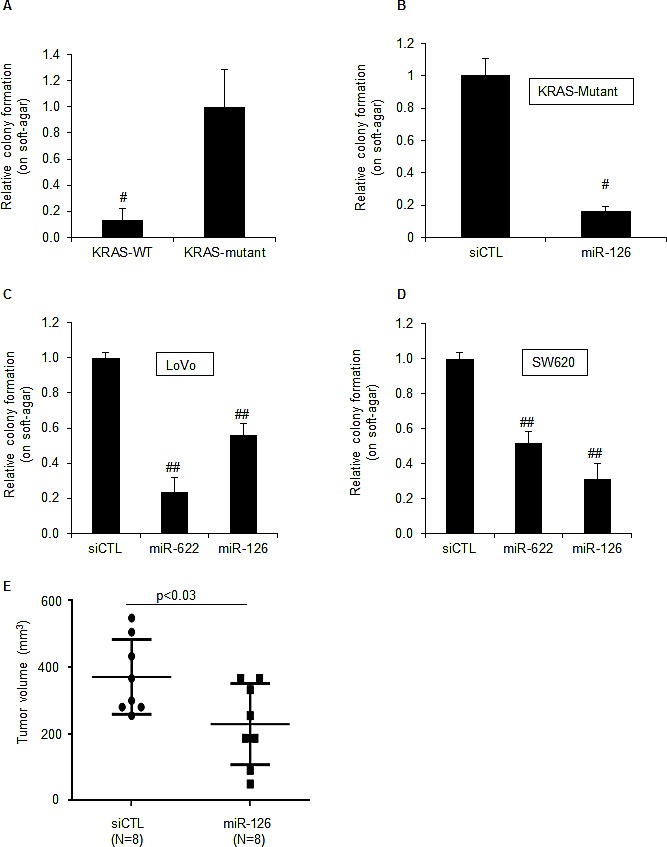
MiR-126 inhibits tumorigenicity of KRAS mutant cells *in vitro* and *in vivo* (A) The tumorigenicity of isogenic HCT116 KRAS-WT and KRAS-Mutant cells was assessed by soft agar colony formation assays. One thousand cells were cultured on soft agar for 3 weeks and the number of colonies was counted. (B) HCT116 KRAS-Mutant cells were reverse transfected with siCTL or miR-126 mimics. After 48 h, 1000 cells were cultured on soft agar for 3 weeks and number of colonies was counted. (C and D) The effect of miR-126 over-expression on colony formation on soft agar for the KRAS-Mutant cells LoVo (C) and SW620 (D) was assessed as described in (B). (E) The effect of miR-126 on tumorigenicity *in vivo* was assessed in mouse xenografts. HCT116 KRAS-Mutant cells were reverse transfected with siCTL or miR-126 mimics. After 48 h, 1×10^6^ cells were mixed with matrigel, injected into the flanks of mice and tumor volume was measured after 9 days. Error bars represent mean ± standard deviation from 3 independent experiments. *, p<0.05; #, p<0.01; **, p<0.005; ##, p<0.001.

We next examined the effect of miR-126 over-expression on tumorigenicity *in vitro* by determining the ability of the cells to form colonies on soft agar 3 weeks after transfection. As previously reported [30, 37], KRAS-Mutant cells formed colonies on soft agar whereas KRAS-WT cells did not (Figure 3A). However, over-expression of miR-126 substantially inhibited (>5-fold) clonogenicity (Figure 3B). To make sure that the reduction in colony formation was not restricted to HCT116-KRAS-Mutant cells, we assessed the effect of miR-126 over-expression on clonogenicity of two additional KRAS-Mutant CRC lines, LoVo and SW620 (Figure 3C and 3D). MiR-126 over-expression significantly impaired the ability of these 2 cell lines to form colonies on soft agar. As a positive control, the KRAS-targeting miR-622 reduced clonogenicity of both lines (Figure 3C and 3D). To test if the inhibition of tumorigenicity by miR-126 also occurs *in vivo*, we transfected HCT116 KRAS-Mutant cells with siCTL or miR-126 mimics for 48 h and generated xenografts in athymic nude mice; tumor volumes were assessed after 9 days. Decreased tumor growth (>40%) was observed in xenografts formed from miR-126 transfected cells (p<0.03; Figure 3E). These results suggest that miR-126 over-expression selectively suppresses the growth of KRAS-Mutant cells both *in vitro* and *in vivo*.

### A subset of miR-126 repressed genes are synthetically lethal with KRAS-mutation

**Figure 4:**
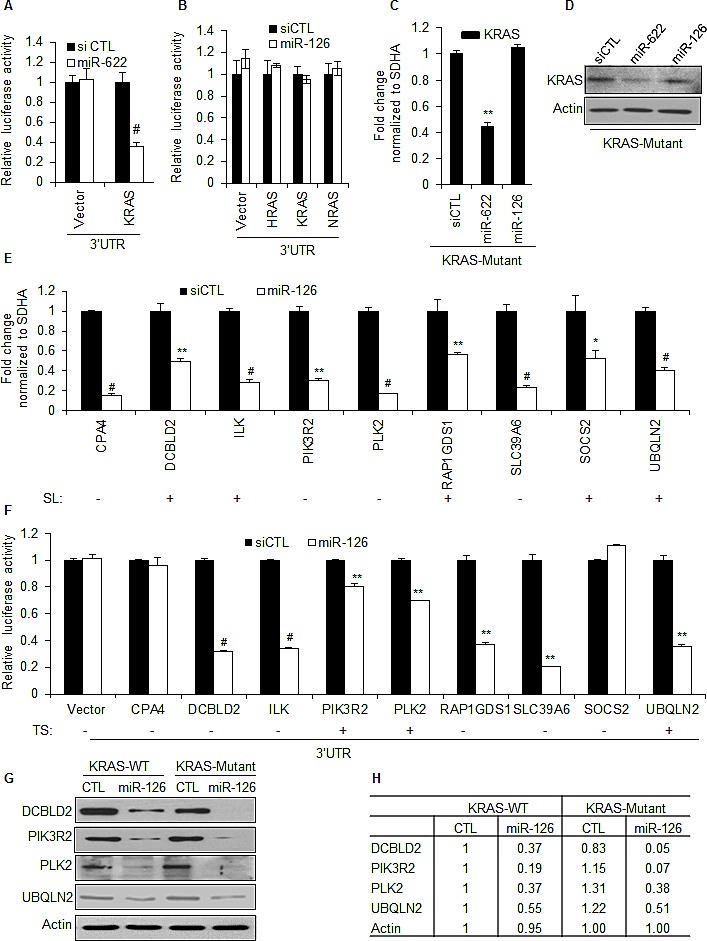
MiR-126 inhibits the 3'UTR of multiple genes selectively required for the viability of KRAS mutant cells (A and B) Luciferase assays were performed from HCT116 KRAS-Mutant cells cotransfected for 48 h with psiCHECK2 (Vector), psiCHECK2 containing the 3'UTR of *KRAS, HRAS* or *NRAS* mRNAs*,* and siCTL, or miR-126 mimics. (C) HCT116 KRAS-Mutant cells were transfected with siCTL, miR-622 or miR-126 mimics for 48 h and RT-qPCR was performed to measure KRAS mRNA levels normalized to the housekeeping gene SDHA. (D) HCT116 KRAS-Mutant cells were reverse transfected with siCTL, miR-622 or miR-126 mimics for 48 h and KRAS protein levels were measured by immunoblotting. Actin was used as loading control. (E) HCT116 KRAS-Mutant cells were reverse transfected with siCTL or miR-126 mimics for 48 h and select miR-126 target mRNAs identified by microarrays were validated by RT-qPCR. Genes previously identified in synthetic lethal RNAi screens for KRAS-Mutant are designated as SL. (F) Luciferase assays were performed from HCT116 KRAS-Mutant cells cotransfected with psiCHECK2 (Vector), psiCHECK2 containing the 3'UTR of miR-126-regulated genes (*CPA4, DCBLD2, ILK, PIK3R2, PLK2, RAP1GDS1, SLC39A6, SOCS2 or UBQLN2*) and siCTL or miR-126 mimics. (G, H) Effect of miR-126 over-expression on protein levels of select miR-126 target genes was assessed by immunoblotting (G); magnitude of down-regulation at the protein level was quantitated by densitometry (H). Error bars represent mean ± standard deviation from 3 independent experiments. *, p<0.05; #, p<0.01; **, p<0.005.

To examine the molecular mechanism(s) by which miR-126 inhibits the growth of KRAS-Mutant cells, we first tested the effect of miR-126 over-expression on a luciferase reporter fused to the 3'UTR of either *KRAS, HRAS*, or *NRAS*. As previously reported [32], the *KRAS*-targeting miRNA, miR-622, significantly inhibited luciferase activity from the *KRAS* 3'UTR (Figure 4A). However, miR-126 did not repress the 3'UTR of *HRAS*, *KRAS*, or *NRAS* mRNAs (Figure 4B). Recently, we and others have shown that miRNAs can also inhibit gene expression by binding to the coding region or the 5'UTR of target mRNAs [38-41]. Therefore, we tested the effect of miR-126 over-expression on endogenous *KRAS* mRNA and KRAS protein levels. MiR-126 did not alter the abundance of *KRAS* mRNA or KRAS protein as measured by RT-qPCR and immunoblotting (Figure 4C and 4D). As expected [32], miR-622 down-regulated KRAS mRNA and KRAS protein (Figure 4C and 4D).

MiR-126 exerted a growth suppressive effect in KRAS-Mutant but not KRAS-WT cells without altering KRAS expression. We therefore hypothesized that miR-126 exerts a context-dependent effect in KRAS-Mutant cells by down-regulating select genes involved in activated KRAS-Mutant signaling. To test this hypothesis, we sought to identify miR-126-regulated genes on a genome-wide scale in both HCT116 KRAS-WT and KRAS-Mutant cells. Recent studies have shown that a large proportion of miRNA target genes can be identified by gene expression profiling after modulating miRNA levels [5, 42-44]. Therefore, we performed microarray analysis from HCT116 KRAS-WT and KRAS-Mutant cells transfected with siCTL or miR-126 mimics (20 nM) for 48 h. Using arbitrary cut-offs of 2.0- or 1.5-fold, respectively (p<0.05), 69 and 321 genes were down-regulated by miR-126 in HCT116 KRAS-Mutant cells, respectively (Table S3). In HCT116 KRAS-WT cells, 66 and 318 mRNAs were down-regulated by miR-126 using cut-offs of 2.0- or 1.5-fold, respectively (p<0.05) (Table S4). The vast majority (218) of miR-126 target genes were down-regulated at least 1.5-fold in both HCT116-KRAS-WT and KRAS-Mutant cells. Approximately 100 genes appeared to be down-regulated only in HCT116-KRAS-WT or KRAS-Mutant cells upon miR-126 over-expression with a cut-off of 1.5-fold. However, a closer examination of the magnitude of the change for these genes indicated that they were also down-regulated in KRAS-WT cells, albeit to a smaller extent (i.e. between 1.3- and -1.5-fold). For instance, BCL9 and STK4 were down-regulated 1.6- and 1.78-fold in the microarrays from KRAS-Mutant cells but the fold change for these 2 genes was 1.38 and 1.42, respectively, in KRAS-WT cells.

Transcriptome profiling did not identify genes down-regulated by miR-126 only KRAS-WT or KRAS-Mutant cells. Therefore, we hypothesized that a subset of miR-126 down-regulated genes may be essential for the viability of KRAS-Mutant but not KRAS-WT cells. To investigate this, we first looked at the overlap of genes that miR-126 down-regulated at least 1.5-fold and the genes previously identified by RNAi screening as synthetically lethal in KRAS-Mutant cells [26]. This strategy identified 22 genes in the intersection of these 2 gene lists (Table S5). For further analysis, we selected 9 genes, *CPA4*, *DCBLD2*, *ILK*, *PIK3R2*, *PLK2*, *RAP1GDS1*, *SLC39A6*, *SOCS2* and *UBQLN2*, that were down-regulated by miR-126 in both KRAS-WT and KRAS-Mutant cells, 5 of which (*DCBLD2*, *ILK*, *RAP1GDS1*, *SOCS2* and *UBQLN2*) were also identified as selectively required for the viability of KRAS-Mutant cells in the synthetic lethal RNAi screen. We first validated that miR-126 over-expression in HCT116 KRAS-Mutant cells down-regulated the expression of these genes by RT-qPCR (Figure 4E). In the isogenic HCT116 KRAS-WT cells, these genes were also down-regulated by miR-126 to the same extent (data not shown). Next, we assessed if these genes were directly regulated by miR-126 using 3'UTR luciferase reporters. Over-expression of miR-126 significantly repressed the 3'UTR of 7 out of the 9 genes (Figure 4F). Interestingly, only three out of these seven 3'UTRs were predicted by TargetScan suggesting that four out of the seven 3'UTRs may be regulated by non-canonical binding sites that can be identified by computational methods such as RNA22 and RNAHybrid because these algorithms do not enforce the seed constraint on miRNA target prediction [3, 45-47]. Additionally, miR-126 reduced protein levels of DCBLD2, PIK3R2, PLK2 and UBQLN2 in both HCT116-KRAS-WT and KRAS-Mutant cells, consistent with these genes being *bona fide* gene targets of miR-126 (Figure 4G and 4H). Among these 4 genes, *PIK3R2* is a known miR-126 target and is predicted by TargetScan, RNA22 and RNAHybrid [48-50]. *PLK2* and *UBQLN2* are also predicted by these algorithms, but have not been previously identified as miR-126 targets. Although *DCBLD2* mRNA does not contain sites complementary to the miR-126 seed sequence and is not predicted by TargetScan, its 3'UTR was repressed by miR-126, indicating that it may be regulated by non-canonical miR-126 binding sites that can be identified by RNA22 or RNAHybrid. Because PIK3R2 is the regulatory subunit of PI3-kinase, we measured AKT phosphorylation upon miR-126 over-expression. However, unlike previous reports that suggested altered AKT phosphorylation upon miR-126 knockdown or over-expression [48, 51, 52], we did not observe a difference in AKT phosphorylation upon transfection of miR-126 mimics in the isogenic lines (Figure S6) suggesting that regulation of AKT phosphorylation by miR-126 may be cell-type specific.

### Silencing miR-126 target genes selectively inhibits the clonogenicity of KRAS-Mutant cells

**Figure 5:**
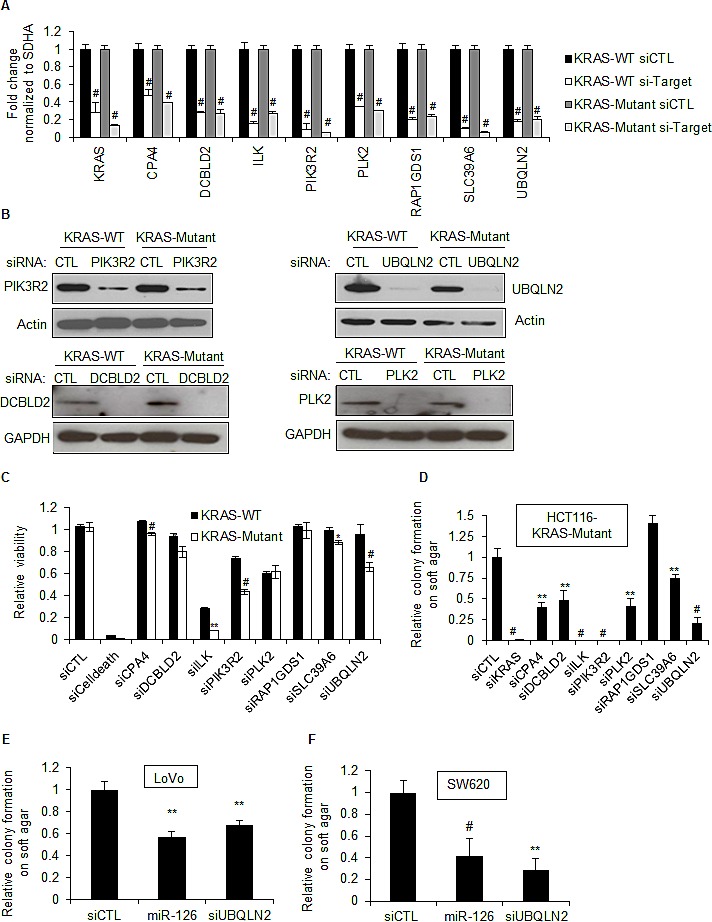
Silencing select miR-126 target genes in KRAS-Mutant cells inhibits clonogenicity (A) The mRNA levels of select miR-126 down-regulated genes were measured by RT-qPCR from isogenic HCT116 KRAS-WT and KRAS-Mutant cells. Isogenic HCT116-KRAS-WT and KRAS-Mutant cells were reverse transfected with siCTL, KRAS siRNAs or siRNAs against miR-126 target genes for 48 h and gene silencing was measured by RT-qPCR. (B) Knockdown of four miR-126 target genes at the protein level was measured by immunoblotting using Actin or GAPDH as loading control. (C) HCT116 KRAS-WT and KRAS-Mutant cells were reverse transfected with siCTL, siCelldeath or siRNAs corresponding to select miR-126 target genes, for 48 h and cell viability was measured by CellTiter-Glo. (D) HCT116 KRAS-Mutant cells were reverse transfected with siCTL, *KRAS* siRNAs or siRNAs corresponding to select miR-126 target genes. After 48 h, 1000 cells were cultured on soft agar for 3 weeks and number of colonies was counted. (E and F) The KRAS-Mutant LoVo and SW620 cells were reverse transfected with siCTL, miR-126 mimics or siUBQLN2 and soft agar colony formation assays were performed as described (D). Error bars represent mean ± standard deviation from 3 independent experiments. *, p<0.05; #, p<0.01; **, p<0.005; ##, p<0.001.

To further examine the context-dependent effect of miR-126, we performed siRNA mediated RNAi against 7 of the confirmed miR-126 target genes, *DCBLD2*, *ILK*, *PIK3R2*, *PLK2*, *RAP1GDS1*, *SLC39A6*, and *UBQLN2*. Although *CPA4* 3'UTR was not repressed by miR-126, we included *CPA4* in this analysis as this was the most strongly down-regulated gene in the microarrays following over-expression of miR-126. We first confirmed siRNA mediated down-regulation of each of these genes by RT-qPCR (Figure 5A). Knockdown at the protein level was also validated for PLK2, PIK3R2, UBQLN2 and DCBLD2 (Figure 5B). We next examined the effect of silencing each of these genes in HCT116 KRAS-WT and KRAS-Mutant cells on cell viability and soft agar colony formation. Only the silencing of *ILK*, *PIK3R2*, and *UBQLN2* resulted in a greater reduction (<40%) in the viability of HCT116 KRAS-Mutant cells as compared to KRAS-WT cells 72 h after transfection, though the silencing of *ILK* also substantially reduced the viability of KRAS-WT cells (Figure 5C). In contrast, silencing of all but one of the selected genes reduced clonogenicity of the KRAS-Mutant cells, in some cases to nearly the same level as observed with KRAS siRNA. The potent inhibition of clonogenicity upon knockdown of these genes in KRAS-Mutant cells indicates that a subset of miR-126 gene targets including *DCBLD2*, *PIK3R2*, *SLC39A6*, and *UBQLN2* may be important for the viability and clonogenicity of KRAS-Mutant cells.

*UBQLN2* was one of the genes whose knockdown selectively reduced the viability of HCT116 KRAS-Mutant cells and also resulted in a highly significant decrease in the colony forming ability of HCT116 KRAS-Mutant cells. *UBQLN2* encodes Ubiquilin-2, a member of the ubiquilin family of proteins that are involved in linking the ubiquitination machinery to the proteasome to effect protein degradation. To determine that the dependency of KRAS-Mutant cells for UBQLN2 was not restricted to HCT116 cells, we transfected the KRAS-Mutant CRC lines LoVo and SW620 with siCTL, *UBQLN2* siRNA, or miR-126 mimics for 48 h and performed soft agar colony formation assays, assessing colony formation after 3 weeks. Silencing *UBQLN2* or over-expressing miR-126 significantly inhibited clonogenicity of the KRAS-Mutant LoVo and SW620 cells (Figure 5E and 5F). Moreover, when we silenced *UBQLN2* in a panel of CRC lines that expressed KRAS-WT or KRAS-Mutant, we observed reduction in the viability of the KRAS-Mutant lines but not KRAS-WT lines (Figure S7A). Consistent with our microarray results where we observed down-regulation of *UBQLN2* mRNA in both KRAS-WT and KRAS-Mutant, we found that over-expression of miR-126 down-regulated *UBQLN2* mRNA in the CRC cell line panel, irrespective of KRAS mutation status (Figure S7B). These results suggest that the miR-126 target gene *UBQLN2* plays a role in the tumorigenicity of KRAS-Mutant cells in a context-dependent manner. Further studies are required to understand the mechanism by which *UBQLN2* regulates the viability of KRAS-Mutant cells. Taken together, our study suggests that by analyzing gene expression changes upon miRNA over-expression and the hits in RNAi-screens, key miRNA-regulated effector genes can be identified to provide mechanistic insights underlying the phenotype regulated by a miRNA.

## DISCUSSION

Although each miRNA can regulate the expression of hundreds of genes, the effect of cellular context on the genes a miRNA regulates is not fully understood. Identifying context-dependent miRNAs and understanding the molecular mechanisms underlying their context-dependent effects will be of fundamental importance to the development of miRNA-based therapeutics targeting the unique genetic and epigenetic features of cancer. In this study, we identified several miRNAs whose over-expression resulted in greater toxicity in KRAS-Mutant cells as compared to KRAS-WT cells. We focused on miR-126, a miRNA down-regulated in colorectal cancer [53, 54] and found that its expression was significantly lower in KRAS-Mutant tumors as compared to tumors expressing WT KRAS. In a pair of isogenic HCT116 cell lines, miR-126 induced potent phenotypic changes in KRAS-Mutant cells not seen in KRAS-WT cells, suggesting that miR-126 is a context-dependent miRNA. Importantly, the context-dependent effect of miR-126 was not restricted to HCT116 cells and was also observed in a panel of CRC lines expressing mutant KRAS. Our results indicate that the context-dependent effect of miR-126 is mediated by suppression of multiple genes essential for the viability of KRAS-Mutant cells but not KRAS-WT cells.

MiR-126 is embedded in an intron of *EGFL7* and is expressed at high levels in vascular tissues such as heart, liver and lung. The expression of miR-126 and its host gene is reduced in most cancers including those with a high frequency of KRAS mutations such as colorectal cancer, lung cancer and pancreatic ductal carcinoma [55]. Recent studies have suggested a tumor suppressor role for miR-126, especially in CRC. MiR-126 regulates diverse cancer-related processes including inflammation, angiogenesis, viability, survival, cell migration and invasion. A number of miR-126 target genes have been reported including *ADAM9*, *CRK, CXCR4, EGFL7*, *HOXA9*, *IRS1*, *KRAS, PIK3R2*, *SOX2* and *SLC7A5, TOM1* and *VEGFA* [48, 51, 52, 54-61]. Within this gene list, *ADAM9*, *CRK, EGFL7*, *IRS1, PIK3R2*, and *SLC7A5* are predicted miR-126 gene targets and were down-regulated by miR-126 in the isogenic KRAS-WT and KRAS-Mutant cells. Interestingly, in a recent study miR-126 was found to inhibit KRAS expression via a seedless binding site in the KRAS 3'UTR in pancreatic cancer cell lines [62]. However, in our study, over-expression of miR-126 did not alter *KRAS* mRNA or protein and had no effect on the *KRAS* 3'UTR. Moreover, KRAS was not identified as a miR-126 target gene in another recent report where microarrays were used to identify mRNAs down-regulated by miR-126 in HT-29 cells (CRC). It is possible that the regulation of KRAS expression by miR-126 may be restricted to pancreatic cancer cells. MiR-126 is a unique miRNA in the sense that its seed sequence is complementary to only a small proportion of the human transcriptome. As a result, TargetScan, a frequently used bioinformatic algorithm for miRNA gene target identification, predicts only 25 conserved gene targets and 154 miR-126 gene targets irrespective of site conservation. Consistent with TargetScan predictions, we found that over-expression of miR-126 reduced the expression of only ~70 genes (2-fold) in KRAS-WT or KRAS-Mutant cells. However, when we reduced the cut-off to 1.5-fold, the number of genes down-regulated by miR-126 (direct plus indirect) was ~300. A substantial proportion of these genes were predicted by algorithms such as RNA22 that do not rely on the presence of a seed sequence in a miRNA target gene. Future studies may identify functional non-canonical miRNA recognition elements in the 3'UTR of these miR-126 target genes by utilizing RNA22.

Given our observation that miR-126 does not target *KRAS* mRNA, we had speculated that some miR-126 gene targets involved in canonical RAS signaling would be down-regulated in KRAS-Mutant cells but not in isogenic KRAS-WT cells. The rationale for this hypothesis was based on recent reports suggesting that RNA-binding proteins modulate the interaction of miRNAs to their targets. For example, HuR assists let-7 to bind to the 3'UTR of *MYC* mRNA[63], Pumulio-1 promotes repression of the 3'UTR of *E2F3* and p27 mRNAs by specific miRNAs[20, 64], and Dnd1 prevents binding of miRNAs to the nanos and tdrd7 mRNAs [65]. If a similar mechanism was involved in the selective targeting of KRAS-Mutant cells by miR-126, we would expect that RNA-binding proteins will block the binding site(s) of miR-126 on some of its target genes involved in canonical RAS signaling in KRAS-WT cells but not in KRAS-Mutant cells, thereby resulting in down-regulation of these genes only in KRAS-Mutant cells. In addition to this mechanism, we had hypothesized the involvement of APA. Multiple recent reports demonstrate that alterations in the length of the 3'UTR due to APA plays a role in the context-dependent effect of some miRNAs such as miR-34a [66], miR-124 and miR-155 [67]. We therefore speculated that due to APA, some miR-126 targets involved in canonical RAS signaling may have shorter 3'UTRs in KRAS-WT cells but not in KRAS-Mutant cells and as a result, miR-126 binding sites in the 3'UTR of these genes would be available for targeting by miR-126 only in KRAS-Mutant cells. In either of these mechanistic scenarios, one would expect selective down-regulation of some miR-126 gene targets between KRAS-WT and KRAS-Mutant cells. Unexpectedly, we did not find a significant difference between the miR-126-regulated transcriptome between KRAS-WT and KRAS-Mutant cells. We believe that our microarrays captured most miR-126 gene targets, based on recent studies suggesting that a majority of genes miRNA target are regulated at the level of mRNA stability [42, 43, 68, 69]. However, we cannot exclude the possibility that some miR-126 target genes are regulated exclusively at the level of mRNA translation and were not identified in our study. Translationally regulated miR-126 gene targets may also be involved in mediating the observed context-dependent effect of miR-126 on KRAS-Mutant cells.

We have utilized a novel strategy for identifying key downstream effectors of miR-126 in KRAS-Mutant cells by comparing the genes down-regulated by miR-126 with the genes identified in a synthetic lethal RNAi screen for KRAS. To our knowledge, this approach has not been used before to identify miRNA effector genes. Our results suggest that the selective effect of miR-126 on KRAS-Mutant cells is mediated by down-regulation of at least five miR-126-regulated genes: *CPA4*, *DCBLD2, PIK3R2, SLC39A6* and *UBQLN2*. Among these five genes, *CPA4*, a gene that was significantly down-regulated in our microarrays, was the only gene whose 3'UTR was not repressed by miR-126. Knocking down these genes individually with siRNAs did not dramatically alter the viability of KRAS-WT or KRAS-Mutant cells over a period of 72 h but resulted in a substantial reduction in clonogenicity of KRAS-Mutant cells. Within this set of miR-126 target genes, Ubiquilin-2 (*UBQLN2*) is a member of the ubiquitin-like protein family. Ubiquilins have an N-terminal ubiquitin-like domain and a C-terminal ubiquitin-associated domain. The ubiquitin-like domain of ubiquilins bind to the proteasome and the ubiquitin-associated domain bind to polyubiquitin chains of proteins [70]. Ubiquilins are therefore thought to functionally link the ubiquitination machinery to the proteasome to induce protein degradation. A recent study identified mutations in ubiquilin-2 as causative of a familial form of ALS (Amyotrophic lateral sclerosis) [71]. Although the proteins UBQLN2 targets have not been identified yet, reporter assays have shown that mutations in UBQLN2 lead to impaired protein degradation [71]. Our results suggest that miR-126 down-regulates *UBQLN2* in both KRAS-WT and KRAS-Mutant cells and this effect is mediated by repressing its 3'UTR. Knockdown of UBQLN2 significantly reduced clonogenicity in three KRAS-Mutant CRC lines, validating our recent study where we found a synthetic lethal interaction between UBQLN2 and mutant KRAS in DLD1 cells [26]. Similar to other genes synthetically lethal to KRAS-Mutant, *UBQLN2* is not known participate in RAS signaling but its expression is apparently necessary for the viability of KRAS-Mutant cells. Given the importance of UBQLN2 mutations in ALS and our results on the selective targeting of KRAS-Mutant cells by miR-126 and upon *UBQLN2* knockdown, it will be important to identify the proteins whose stability is regulated by UBQLN2. Identifying the substrates of UBQLN2 in KRAS-Mutant cells may provide insights onto the role of protein degradation in KRAS-driven oncogenesis. The miR-126 gene targets *CPA4*, *DCBLD2, PIK3R2, SLC39A6* and *UBQLN2* are interesting candidates for future work and it will be important to understand the molecular mechanism by which these genes regulate viability and clonogenicity in the context of mutant KRAS.

By integrating the gene targets of a miRNA with genes identified by loss-of-function RNAi screening it may be possible to identify a subset of genes critical to a phenotype regulated by a context-dependent miRNA. Although our study focused on miR-126, future studies may examine the selective targeting of KRAS-Mutant cells by other miRNAs identified in our miRNA screen. Moreover, future investigations that utilize a combination of unbiased screening approaches and miRNA gene target identification will likely identify miRNAs and their key effectors in other genetic contexts.

## MATERIALS AND METHODS

### Cell culture, siRNA and miRNA reagents

The isogenic colorectal cancer cell lines HCT116 KRAS-WT and HCT116 KRAS-Mutant (KRAS-G13D/−) were previously generated [30] and kindly provided by Bert Vogelstein (Johns Hopkins University, Baltimore, USA). All other cell lines were purchased from ATCC (Manassas, USA) and maintained in Dulbecco's modified Eagle's medium (DMEM) containing 10% fetal bovine serum (FBS) at 37°C with 5% CO_2_. SW403, SW1116 and SW620 were maintained in Leibovitz's L-15 Medium containing 10% fetal bovine serum (FBS) at 37°C. The siRNAs corresponding to cyclophilin B, *KRAS*, the negative control siRNA (siCTL), control miRNA mimics (cel-miR-67), and all miRNA mimics were purchased from Dharmacon/Thermo scientific (Pittsburg, USA). The siCellDeath siRNA and siRNAs targeting *CPA4*, *DCBLD2*, *ILK*, *PIK3R2*, *PLK2*, *RAP1GDS1*, *SLC39A6*, *SOCS2*, and *UBQLN2* mRNAs were purchased from Qiagen (Valencia, USA). All miRNA mimic and siRNA transfections were performed by reverse transfection at a final concentration of 20 nM using Lipofectamine RNAiMAX (Life technologies, Grand Island, USA) as directed by the manufacturer.

### Luciferase reporter assays

The 3'UTRs of *CPA4*, *DCBLD2*, *ILK*, *PIK3R2*, *PLK2*, *RAP1GDS1*, *SLC39A6*, *SOCS2* and *UBQLN2* were amplified by PCR (primer sequences in Table S6) from genomic DNA isolated from HCT116 cells and inserted into the *Renilla* luciferase 3'UTR of psiCHECK2 (Promega, Madison, USA). Luciferase reporter assays were performed in HCT116-KRAS-Mutant cells as previously described [31].

### MiRNA mimic screen

The miRNA mimic screening assays were performed using the Human miRIDIAN miRNA Mimic Library (18.0) purchased from Dharmacon. The library was arrayed in three 384-well plates. Each plate also contained the following controls; 32 wells with no RNA, and 8 wells each of siCTL, siCelldeath, control miRNA mimic, miR-34a mimic, and a siRNA corresponding to PLK1. Transfections were performed by pre-complexing miRNA mimic (0.5 pmol) with 0.1 μl RNAiMAX in 25 μl of serum-free DMEM per well for 15 min at room temperature. Five hundred cells per well were added in 25 μl DMEM supplemented with 20% FBS to yield transfection mixtures consisting of 10 nM miRNA in DMEM with 10% FBS. Plates were maintained at 37°C in a humidified atmosphere containing 5% CO_2_. After 72 h, cell viability was measured using the CellTiter-Glo assay (Promega) following manufacturer's instructions. Two replica screens were preformed one week apart. Quality control data for all control siRNAs and miRNAs, in both screen replicas, is shown in Figure S2B, and the Z-factors for the cell viability assay for each plate calculated using the siCTL and siPLK1 transfected wells is shown in Figure S2C. Data was median normalized and z-score transformed; miRNAs were considered to have a differential effect on the growth of HCT116-KRAS-Mutant versus KRAS-WT cells if the KRAS-Mutant z-score was 0.5 less than that of the KRAS-WT cells.

### RNA isolation, RT-qPCR and miRNA analysis

Total RNA was isolated using TRIzol reagent (Invitrogen) as directed by the manufacturer. For quantitative reverse transcription-PCR (RT-qPCR), 200 ng total RNA was reverse transcribed using iScript Reverse Transcription Supermix (Bio-Rad, Hercules, USA), and qPCR was performed using Fast SYBR Green Master Mix (Life technologies, Grand Island, USA) as described by the manufacturer. Primer sequences are detailed in Table S6. TaqMan miRNA assays were purchased from Life technologies and used to quantitate mature miRNAs following the manufacturer's instructions. *U6* snRNA was used as an internal control. To determine the amount of mature miR-126 associated with Ago2, the isogenic HCT116 lines were transfected with siCTL or miR-126 mimics for 48 h and miR-126 levels measured in the Ago2 IPs from cytoplasmic extracts by RT-qPCR as previously described [31].

### Immunoblotting

Whole-cell lysates were prepared by using radioimmunoprecipitation assay (RIPA) buffer containing protease inhibitor cocktail (Roche, Nutley, USA), and proteins were quantified by using a bicinchoninic acid (BCA) protein quantitation kit (Thermo Scientific). Ten micrograms of whole-cell lysate per lane was used for immunoblotting. The following antibodies were used: anti-KRAS (Sigma-Aldrich, St. Louis, USA), anti-DCBLD2 (Cell Signaling, Danvers, USA), anti-p-AKT-Ser473 (Cell Signaling), anti-p-AKT-Thr308 (Cell Signaling), anti-total-AKT (Cell Signaling), anti-PIK3R2 (Santa Cruz, Dallas, USA), anti-PLK2 (Santa Cruz), and anti-UBQLN2 (Millipore, Billerica, USA) at 1:1000 dilution; anti-β-Actin and anti-GAPDH (Cell Signaling) at a dilution of 1:2000.

### Cell viability and colony formation assays

Cell viability assays were performed after seeding 1000 cells per well of a 96-well plate and using Cell Counting Kit-8 (Dojindo, Rockville, USA) as described by the manufacturer. For colony formation on plastic, cells were transfected with siCTL or miRNA mimics for 48 h, trypsinized and 1000 cells were seeded per well of 12-well plate. After 10 days, the cells were fixed with ice-cold 100% methanol for 5 min, stained with crystal violet (Sigma-Aldrich) and the number of colonies were counted. For soft agar colony formation assays, cells were transfected with a siCTL, *UBQLN2* siRNA or miRNA mimics for 48 h, trypsinized and the number of colonies were counted 3 weeks after growing the cells (1000 cells per well of a 12-well plate) on soft agar.

### Flow cytometry assays

Cells were transfected with siCTL or miR-126 mimics for 72 h, fixed with ice-cold ethanol for 24 h and stained with propidium iodide (Sigma-Aldrich) in the presence of RNase. DNA content was analyzed on a FACSCalibur (BD Biosciences, San Jose, USA).

### Xenograft assays

Animal studies were performed under protocols approved by the National Cancer Institute Animal Care and Use Committee following AALAAC guidelines and policies. Cells were transfected with siCTL or miR-126 mimics for 48 h and then trypsinized and washed with PBS. 1×10^6^ cells were mixed with 30% Matrigel in PBS on ice and the mixture was injected into the flanks of 6-8 week old female athymic nude mice (Animal Production Program, Frederick, MD) (N = 10) each group. Tumor volume was measured 9 days after the injection.

### Analysis of miRNA expression in TCGA

To compare the expression of miR-126, *EGFL7* and miR-132 between CRC patient samples with defined KRAS mutation status, the TCGA data for these RNAs was downloaded from. http://www.cbioportal.org/public-portal. Student's t-test was used to calculate the p-value.

### SUPPLEMENTARY MATERIAL AND FIGURES



### SUPPLEMENTARY MATERIAL AND TABLES


