# Supplementary material for: Selective targeting of KRAS-Mutant cells by miR-126 through repression of multiple genes essential for the survival of KRAS-Mutant cells
Source: Oncotarget. 2014 Jul 31;5(17):7635–50. doi: 10.18632/oncotarget.2284 (PMC4202150; doi:10.18632/oncotarget.2284)
Supplement: Supplementary file 1 [file oncotarget-05-7635-s001.pdf]

Selective targeting of KRAS-Mutant cells by miR-126 through repression of multiple genes essential for the survival of KRAS-Mutant cells

Supplementary Material

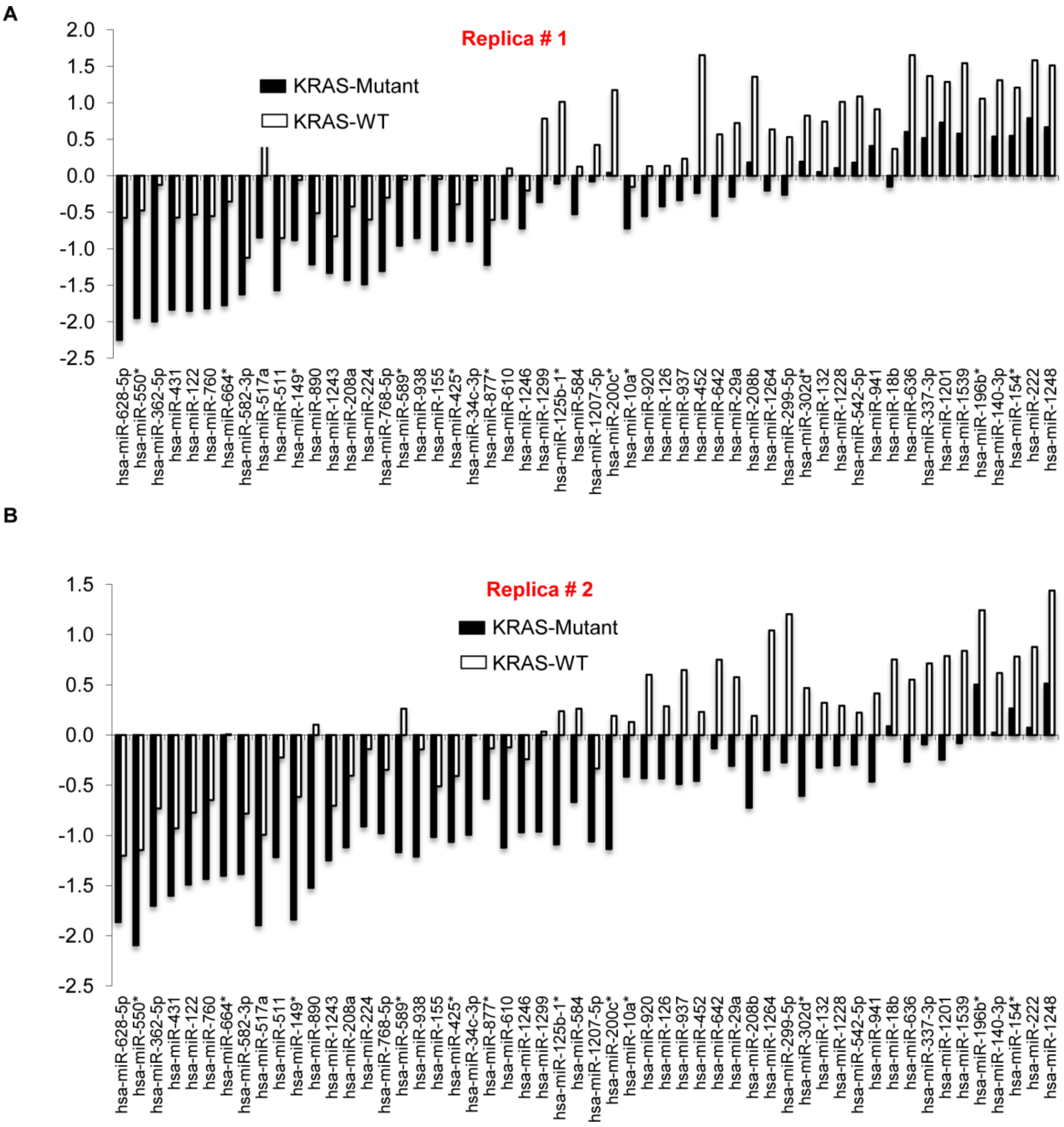

Figure S1: (A, B) Mimics corresponding to 54 miRNAs that induced a difference in the viability of HCT116 KRAS-Mutant compared to KRAS-WT cells 72 h post-transfection (Replica 1 and Replica 2). Cell viability was assayed using CellTiter-Glo and data is shown as median (all miRNAs) normalized z-scores.

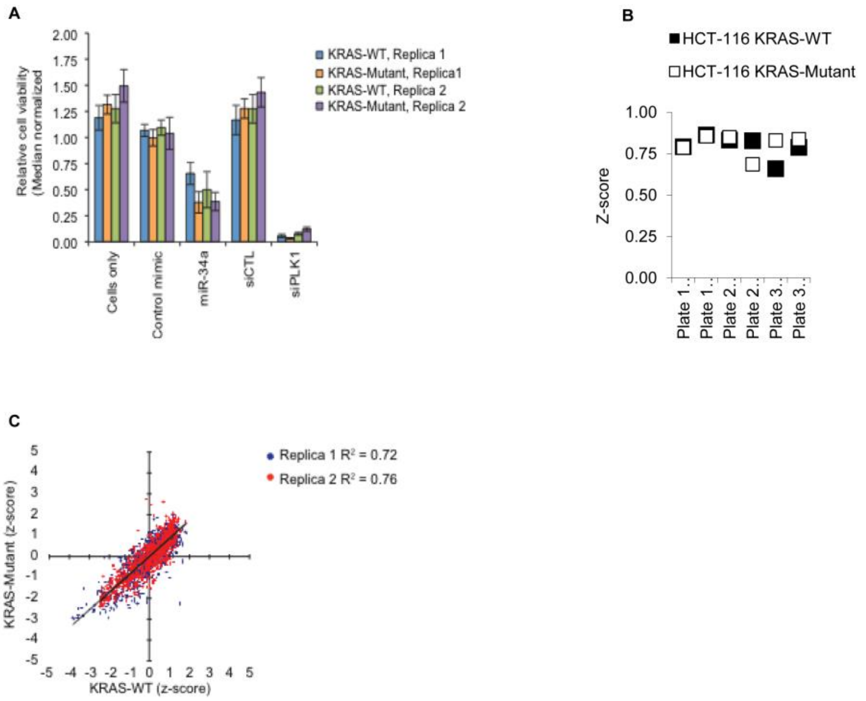

Figure S2: (A) The relative cell viability (median normalized) for control RNAs included in each plate within the replica screens (screens 1 and 2). Data is shown as the mean and standard deviation for 96 untransfected wells (cells only) and 24 transfected wells per RNA (Control mimic RNA, miR-34a, siCTL, and an siRNA corresponding to PLK1 (siPLK1). (B) Z' values calculated for each plate in each screen using the cell viability data obtained for the siCTL and siPLK1 transfected wells. A Z' of over 0.5 represents an excellent assay. (C) The correlation of the effects of each miRNA mimic on the relative viability of HCT116 KRAS-WT and KRAS-Mutant cells for both screen replicas.

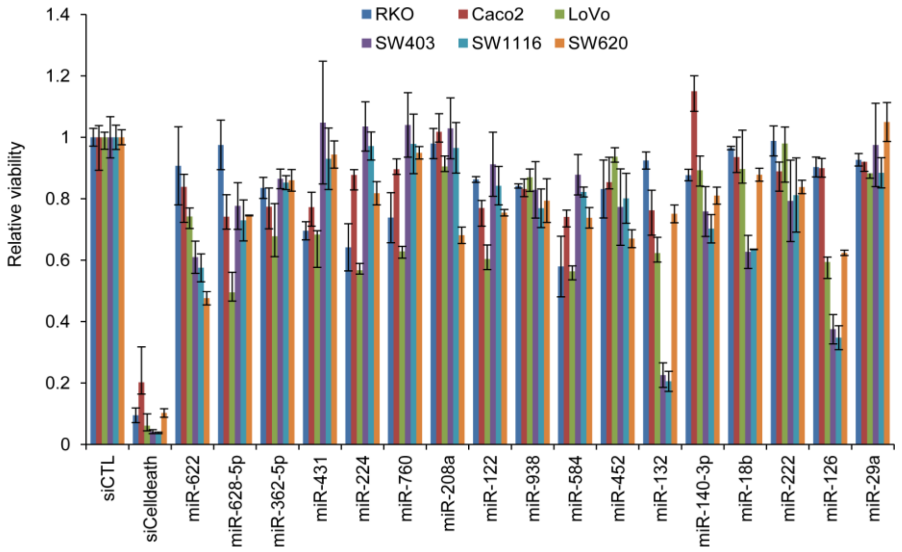

Figure S3: Effect of over-expression of 16 miRNA mimics on the viability of a panel of CRC lines was assessed. These miRNAs selectively impaired the viability of HCT116 KRAS-Mutant cells, as assessed by the miRNA mimic screening results.

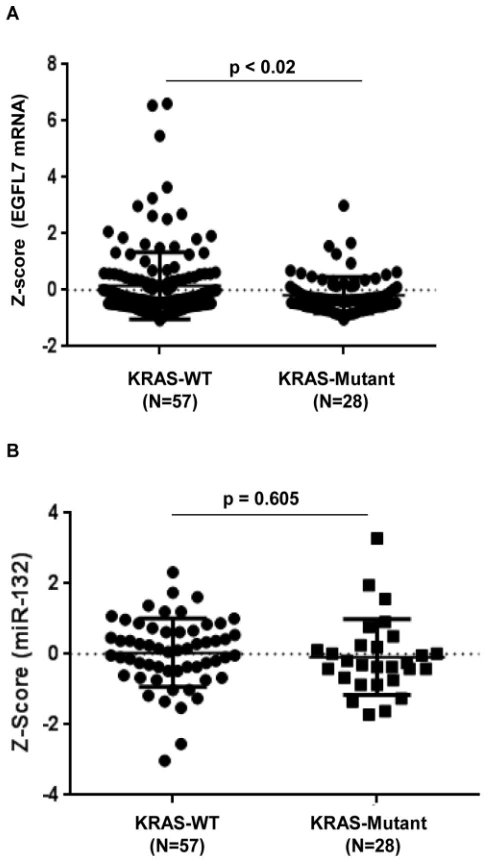

Figure S4: (A and B) The levels of EGFL7 mRNA and miR-132 between KRAS-WT and KRAS-Mutant CRC patient samples (TCGA/cBioPortal) are shown (see Table S2 for Z-scores for all samples).

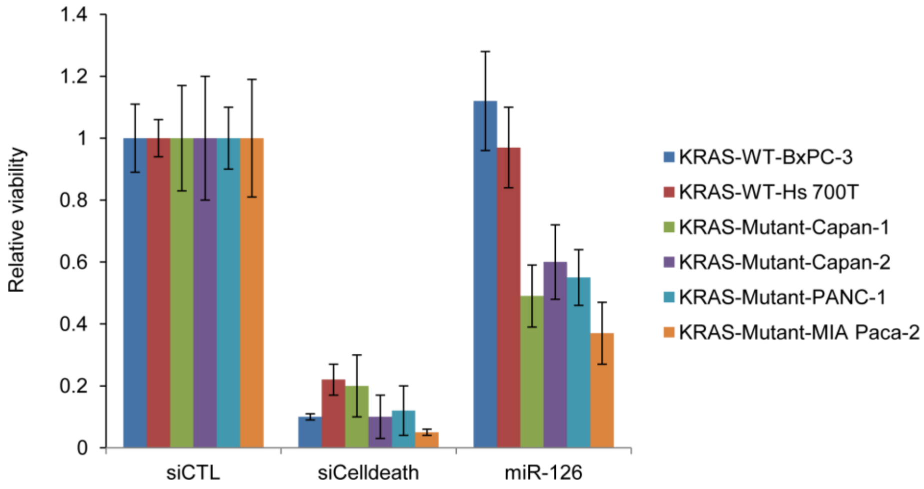

Figure S5: Effect of miR-126 over-expression on the viability of a panel of pancreatic cancer cell lines was examined. KRAS-WT (BxPC-3 and Hs 700T) or KRAS-Mutant (Capan-1, Capan-2, PANC-1 and MIA Paca-2) cells were reverse transfected with siCTL, siCelldeath or miR-126 mimics and cell viability assays were performed after 72 hr.

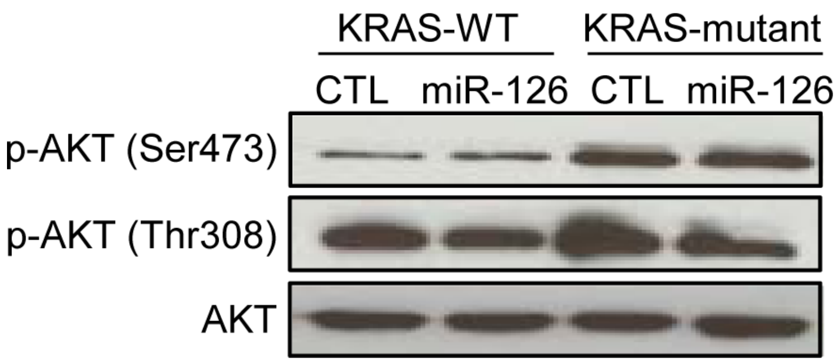

Figure S6: Effect of miR-126 over-expression on AKT phosphorylation was assessed by immunoblotting from isogenic HCT116 KRAS-WT and KRAS-Mutant cells, 48 h after transfection with siCTL or miR-126 mimics. Actin was used as loading control.

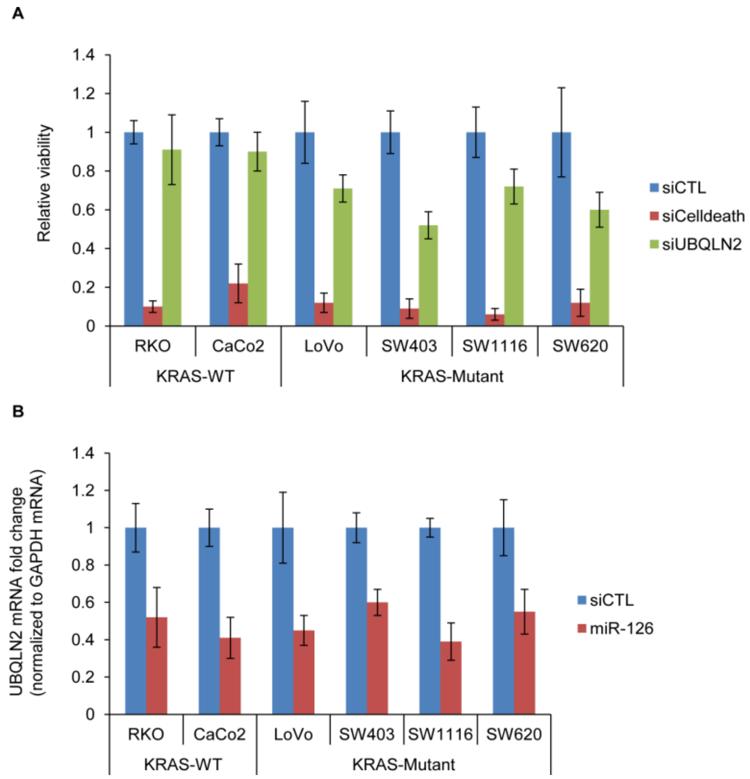

Figure S7: (A) The effect of miR-126 over-expression on the mRNA levels of the miR-126 target gene UBQLN2 was determined by RT-qPCR after transfecting siCTL or miR-126 mimics in KRAS-WT (RKO and CaCo2) or KRAS-Mutant (LoVo, SW403, DW116 and SW620) CRC cells for 48 hr. (B) Effect of silencing UBQLN2 on the viability of a panel of KRAS-WT or KRAS-Mutant CRC lines was measured by cell viability assays.
